# Fabrication of robust and durable superamphiphobic aluminum alloy and zinc surfaces via dual sandblasting and steam treatment

**DOI:** 10.1007/s00170-025-16891-z

**Published:** 2025-11-11

**Authors:** Laylan B. Hassan, Nawzat S. Saadi, Tansel Karabacak

**Affiliations:** 1 Department of Physics and Astronomy, University of Arkansas at Little Rock, Little Rock, AR 72204 USA; 2 Department of Physics, University of Duhok (UoD), Duhok, 1006 AJ Iraq

**Keywords:** Superamphiphobic surfaces, Sandblasting, Steam treatment, Hierarchical structures, Mechanical durability

## Abstract

We present a scalable and environmentally friendly method for fabricating mechanically robust superamphiphobic coatings on aluminum alloy and zinc substrates using a dual-step process combining sandblasting (SB) and steam treatment (ST), followed by surface energy reduction with fluorinated molecules. This approach creates hierarchical micro/nano structures essential for omniphobic performance. On Al-alloy SB + ST surfaces we measured static contact angles of 162.0° (water), 156.1° (ethylene glycol), and 154.4° (peanut oil), while the corresponding Zn surfaces reached 160.1°, 156.0°, and 152.8°, respectively, with sliding angles below 5° across all tested liquids. The coatings retained high repellency after 50 tape-peeling cycles and 100 cm of sandpaper abrasion under a 500 g load (e.g., ethylene glycol > 140° and peanut oil ≈ 120°). They also showed resistance to water jet impact, excellent self-cleaning, and anti-fogging performance. Compared to conventional hot water treatment or chemical etching, this ST-based method enables faster, cleaner fabrication and significantly enhances mechanical durability making it a promising candidate for large-scale applications in anti-fouling, anti-corrosion, and protective surface technologies.

## Introduction

In nature, many surfaces such as lotus leaves, water strider legs, and cicada wings exhibit excellent wetting behavior as reflected by their high capacity to repel water [1]. Such natural superhydrophobic systems have high water contact angles (CA > 150°) and low sliding angles (SA), so that water droplets can move freely off and remove the dirt, exhibiting self-cleaning ability [2]. Following these biological surfaces, significant work has been performed to create artificial superhydrophobic surfaces through methods such as chemical etching [3, 4], sol-gel processing [5], electrospinning [6], nanoparticle deposition [7], and anodization [8] techniques. A further extension of this concept is superamphiphobicity, whereby the surface not only repels water but also low-surface-tension fluids such as oils [9]. Superamphiphobic surfaces exhibit contact angles of more than 150° for a wide range of liquids and have attracted growing interest due to their potential in application in self-cleaning, anti-corrosion [10], anti-fouling [11], anti-icing [4], and drag reduction [12]. However, in contrast to superhydrophobic surfaces, it is harder to fabricate such surfaces since, aside from having very low surface energy, it must also possess a specially engineered surface structure able to resist low-surface-tension fluid intrusions like hexadecane (surface tension ~ 27.5 mN/m at 20 °C).

Superamphiphobicity is achieved by fabricating hierarchical micro/nanostructures and surface functionalization with low-surface-energy materials. In spite of the achievements that have been made so far in developing superhydrophobic Al alloy surfaces via a wide range of disparate approaches namely, chemical etching [3, 13], sol-gel deposition [14, 15], anodization, hot water treatment (HWT) [16–22] and fluorosilane treatment the successful development of superamphiphobic aluminum surfaces has not been widely reported. Among the various hydrothermal oxidation techniques used to fabricate nanoscale features on metal surfaces, HWT and ST are two commonly employed methods.

ST can be considered a derivative of HWT, offering an alternative route to form metal oxide nanostructure on metal surfaces. In our previous work, we employed HWT in combination with sandblasting to develop hierarchical structures essential for surface functionality [17, 18]. Building on that approach, ST provides a comparable method for metal oxide nanostructure formation while maintaining a similarly simple, chemical-free, and scalable process for engineering textured metal surfaces. Compared to traditional fabrication routes like chemical etching [23–25], anodization [26], or electrochemical treatments [27], many of which suffer from long processing times, low durability, or limited oil repellency, ST offers a cleaner and more broadly applicable alternative.

However, despite extensive efforts, most reported superhydrophobic or superamphiphobic metal surfaces still face critical challenges, including limited mechanical durability, complex or hazardous chemical processing, and poor scalability for industrial applications [28, 29]. These drawbacks restrict their use in real-world environments that require both high liquid repellency and long-term robustness. Therefore, there is a clear need to develop a simple, chemical-free, and environmentally friendly method capable of producing mechanically stable superamphiphobic coatings on metallic substrates [30, 31].

In this study, we introduce a simple and efficient method for fabricating mechanically robust superamphiphobic surfaces on zinc and aluminum alloy substrates using a two-step process combining sandblasting and ST. The sandblasting step generates microscale roughness, while ST induces the growth of nanoscale features, together forming the hierarchical surface morphology essential for superamphiphobic behavior. Subsequent chemical functionalization with a fluorinated compound renders the surfaces highly repellent, exhibiting static contact angles above 160° and sliding angles near 0° for both water and oil-based liquids.

The primary objectives of this study are (i) to establish a dual-step sandblasting and steam treatment process for creating hierarchical micro/nano structures on aluminum alloy and zinc surfaces. (ii) to evaluate the resulting surfaces in terms of their wetting, mechanical, and self-cleaning performances, and (iii) to demonstrate that this process can provide an efficient and scalable route toward durable, environmentally benign superamphiphobic coatings.

The resulting surfaces also demonstrate excellent self-cleaning, anti-fogging, and mechanical durability under adhesion and abrasion tests. This scalable and environmentally benign approach offers a promising route for developing superamphiphobic coatings for industrial applications.

In this context, the present study targets aluminum alloy and zinc substrates because of their widespread industrial relevance and the need for durable coatings suitable for these materials. The proposed dual-step sandblasting and steam treatment method directly addresses the shortcomings of previous approaches by combining a clean, chemical-free process with the creation of hierarchical structures critical for sustaining superamphiphobicity. Furthermore, comparative insights from the literature underline that this process achieves superior durability and wetting resistance relative to conventional hydrothermal and etching techniques.

## Materials and methods

### Fabrication of Multi-Scale rough surfaces on aluminum alloy and zinc substrates

Superamphiphobic surfaces with nano- and hierarchical micro/nano-scale roughness were fabricated using commercial Al-clad (2024 T3) and zinc substrates. The substrates were cut into uniform 30 mm × 30 mm samples. Microscale roughness was introduced by sandblasting with aluminum oxide (Al₂O₃, 120 mesh) under 4.5 Pa compressed air pressure [18]. After sandblasting, samples were ultrasonically cleaned in isopropanol and deionized water for 10 min each to remove residual particles. For control samples, the native oxide layers were removed using ultra-fine 5000-grit sandpaper, followed by the same ultrasonic cleaning process. Nanoscale roughness was developed using optimized ST: aluminum alloy substrates were treated for 1 h, and zinc substrates for 2 h, enabling the formation of nanostructures essential for superamphiphobicity. Dual-scale (hierarchical) roughness was achieved by applying the same ST to the sandblasted substrates.

**Fig. 1:**
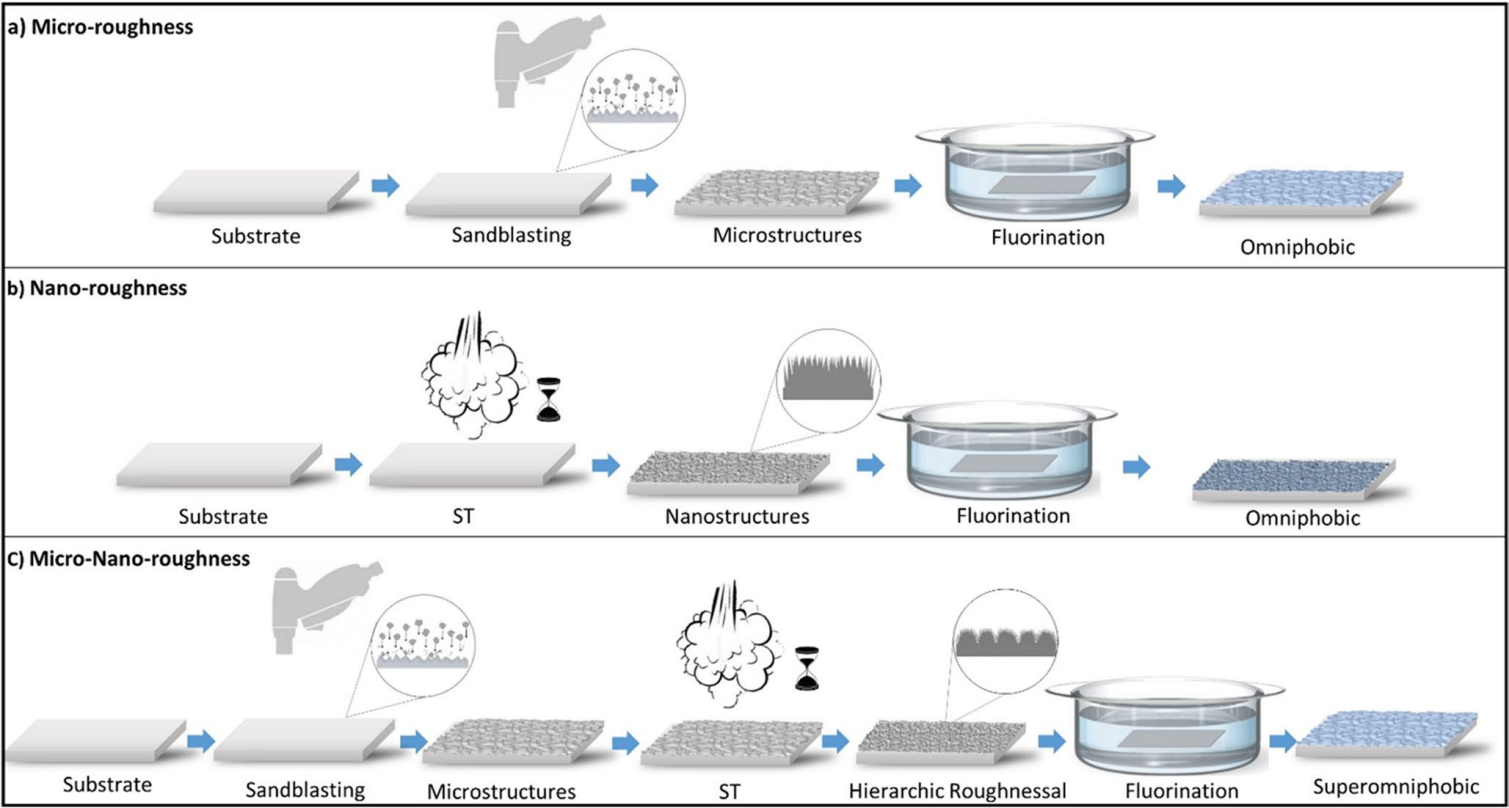
illustrates the step-by-step process for fabricating superamphiphobic surfaces: **a** Sandblasting (SB) is used to create roughness at the microscale, **b** Steam treatment (ST) introduces nanoscale texture, and (**c**) combining SB and ST produces a hierarchical structure featuring both micro- and nano-level roughness. Finally, a fluorination step is applied to lower the surface energy

To impart low surface energy, all surface types smooth, micro-roughened, nano-roughened, and hierarchically roughened were chemically modified using self-assembled monolayers (SAMs). Aluminum alloy substrates were functionalized with perfluorodecyltrichlorosilane (PFDTS) and cured at 90 °C for 3 h. Zinc substrates were immersed in a dodecanethiol (DDT) solution for 10 min. These treatments successfully enhanced both hydrophobicity and oleophobicity, as illustrated in Fig. 1.

### Material and wettability characterization

In order to characterize the surface morphology and wetting behavior of the samples the scanning electron microscopy (SEM), and contact angle measurements were performed. Wetting behavior of the samples were evaluated by contact angle (CA) measurements using a VCA Optima goniometer contact angle instrument at room temperature using the sessile drop fitting method for the static CAs. Liquid droplets of 5 ± 1 µl size of DI water, ethylene glycol and peanut oil were gently distributed on the surfaces, and CAs were measured on more than six different places for the control, micro-, nano-, and micro-nano hierarchical samples surface before and after surface energy reduction (SER). The sliding angles were also measured after SER. Scanning electron microscopy SEM (SEM; JEOL-JSM7000F) analyzed the surface morphology of the samples.

### Surface robustness and durability tests

Superamphiphobic coatings are often susceptible to degradation under harsh mechanical and environmental conditions, such as abrasion, bending, water impact, and corrosive media. To assess the mechanical durability of the fabricated coatings, a series of tests were conducted, including sandpaper abrasion, adhesive tape peeling, water-jet impact, and surface bending. For the abrasion test, each sample was rubbed against 1500-grit sandpaper under a 500 g load at a constant speed of 10 mm/s over a 100 cm distance. The adhesion test involved applying and removing adhesive tape (NW-H25, Nichiban Co. Ltd., Japan) from the coated surfaces to evaluate resistance to surface peeling for 50 cycles. Post-test changes in surface morphology and wettability were examined using SEM and contact angle measurements.

Water-jet impact resistance was evaluated by spraying water at a 35° angle with a velocity of 2.6 m/s. In addition, a floatation test was performed by placing samples on water and peanut oil surfaces and recording the time taken for the sample to submerge, indicating the retention of air pockets and surface repellency.

The self-cleaning ability of the coatings was tested by sprinkling Al₂O₃ abrasive dust (180 mesh) onto the surface, followed by the application of water and oil droplets to observe contaminant removal. Anti-fogging performance was evaluated by exposing the coated surface to steam from boiling water and monitoring its ability to prevent moisture condensation.

These tests collectively demonstrate the coating’s robustness and functionality under realistic mechanical and environmental challenges, supporting its suitability for practical applications on aluminum-based components.

## Results and discussions

### Surface morphology and topography

**Fig. 2:**
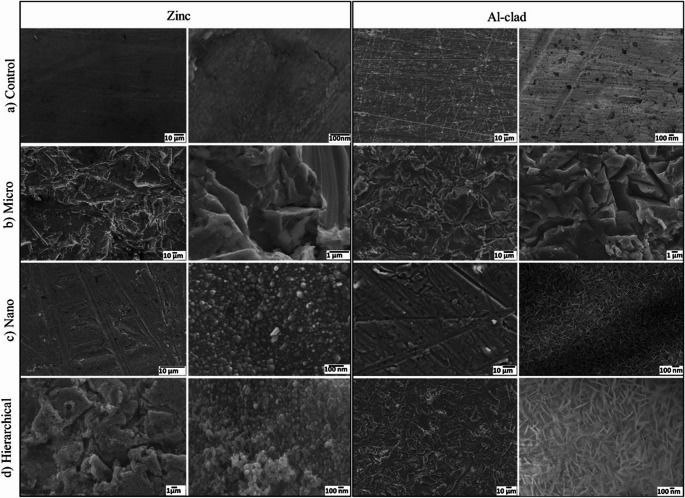
Surface morphology of Zn and Al alloy substrates after different surface treatments as observed by SEM. **a** control samples (sandpapered using 5000-grit sandpaper), showing smooth surfaces. **b** Surfaces after sandblasting (SB), showing micro-scale roughness due to mechanical erosion. (**c**) Steam-treated surfaces forming short nanowires on Zn and dense “nano-grass” nanoscale features on Al allo. **d** is the combined SB + ST treatment, forming hierarchical micro-nano structures required to obtain superamphiphobicity. High-magnification insets highlight the surface texture at sub-micron to nanometer scales

Surface morphology of Al alloy and Zn substrates was systematically characterized by SEM to examine the effect of sandblasting, ST, and combined (sandblasting + ST) treatment with specific focus on the development of hierarchical structures suitable for imparting superamphiphobic properties. Typical SEM images representing morphological evolution among untreated, SB, ST, and SB + ST treated surfaces for both metals are provided in Fig. 2.

The control samples Fig. 2a, having been polished using 5000-grit sandpaper, presented flat and relatively featureless Al alloy and Zn surfaces. The SEM topographies are smooth and feature only the presence of slight abrasive lines, without any hint of micro- or nano-scale structuring. These flat surfaces inherently lack the dual-scale roughness for superamphiphobicity, as they cannot support the requisite composite solid–air–liquid interface for repelling both high- and low-surface-tension liquids.

Upon sandblasting Fig. 2b, clear micro-scale surface features were imparted due to mechanical erosion from high-speed abrasive particles. On Al alloy, this led to irregular, flaky microstructures of the order of several micrometers in height, forming a rough surface texture. Likewise, the Zn substrate formed pronounced striations and fractures, suggesting brittle deformation in response to mechanical stress [18]. These microstructures are necessary for air trapping and establishing the first degree of roughness for liquid repellency. Micro-roughness alone is, however, insufficient to realize superamphiphobicity; features at the nanoscale are also necessary to prevent liquid pinning and allow for low contact angle hysteresis for both water and low surface tension liquids such as oils and alcohols.

Steam treatment alone Fig. 2c formed very unique nano-scale structures with distinct characteristics on each substrate. On the Al alloy surface, ST induced the formation of dense, nano-grass structures. The vertically aligned nanostructures densely covered the surface to form a thin topographic layer with superior ability to resist wetting by polar and non-polar liquids. On the Zn substrate, ST induced the formation of short and compact nanowires (~ 50–100 nm), which were evenly distributed on the relatively smooth background. These nano-textures establish the secondary scale of roughness that is essential to the creation of re-entrant geometries for sustaining a Cassie–Baxter state even under low surface tension liquid exposure.

The hierarchical roughness essential for superamphiphobicity was primarily achieved through the combination of sandblasting and ST (Fig. 2d). On Al alloy, sandblasting created microscale hills and troughs, while subsequent ST produced dense nano-grass features atop this structure. This dual-scale morphology introduces the topographical re-entrance needed to repel low-surface-tension liquids like hexadecane and ethanol. This is attributed to steam’s vapor-phase oxidation, which limits lateral diffusion and promotes localized vertical growth needle-like oxide features.

Similarly, the substrates of Zn under SB + ST presented a dense layer of moderately tilted nanowires (~ 100 nm), anchored in the micro-roughened surface created by sandblasting. These nanowires were significantly shorter and denser than those typically formed under SB + HWT conditions (150–300 nm) [18, 32]. This difference arises from the reduced diffusion rate of ionic species in the vapor phase during steam treatment, which promotes high nucleation density but limits axial growth. The resulting compact nanowire packing on Zn contributes to the hierarchical roughness essential for omniphobic behavior. Moreover, the shorter nanowires reduce the risk of capillary intrusion by low-surface-tension liquids—an important factor for achieving and maintaining superamphiphobicity.

The SEM observation clearly demonstrates that the combined action of sandblasting and ST generates a hierarchical surface structure on both Al alloy and Zn substrates. The integration of mechanically generated micro-roughness and vapor-phase chemically generated nano-roughness results in complex re-entrant features and air-retaining structures, both of which are essential to providing superamphiphobic functionality. Such surfaces can repel a wide range of liquids by minimizing contact area and stabilizing the Cassie state and have excellent potential to be utilized in high-end applications like anti-fouling, self-cleaning, and corrosion-resistant coatings.

### Wetting behavior study of superamphiphobic surfaces

Wetting behavior on the Al alloy and Zn substrates was studied systematically by static contact angle measurements using three model liquids: water, ethylene glycol, and peanut oil, representing high, moderate, and low surface tension liquids, respectively. The aim of this test was to confirm the repellency of the surfaces towards both polar and non-polar liquids a characteristic of superamphiphobicity. A surface is typically referred to as superamphiphobic if it exhibits static contact angles of more than 150° for a broad range of liquids and small contact angle hysteresis or roll-off angles. Across all conditions, the standard deviations (SDs) for static contact angles were low (typically ± 2–3°), indicating good spatial uniformity and repeatability of the wetting response on the textured surfaces.

#### Wetting performance on Al- alloy surfaces

**Fig. 3:**
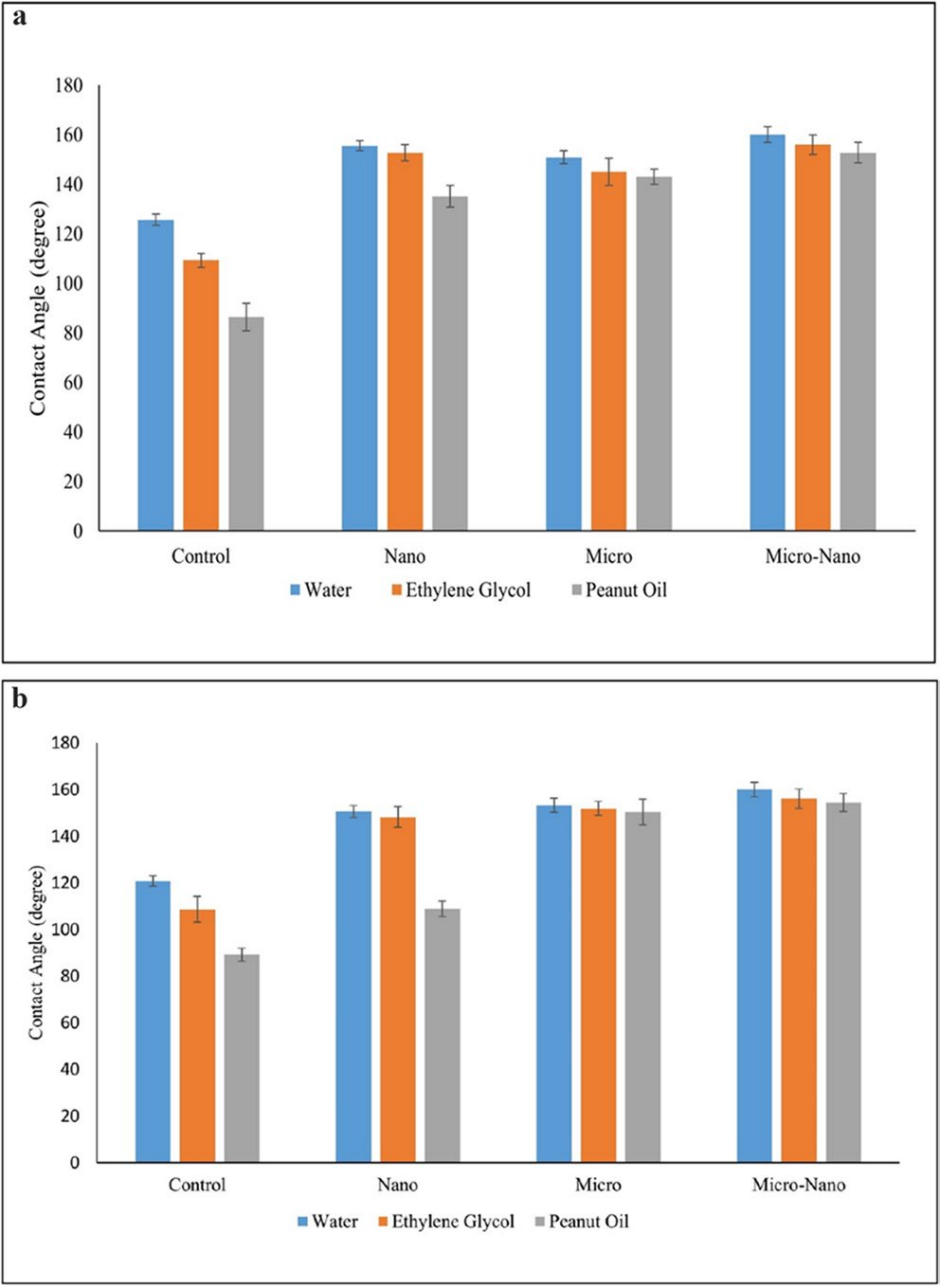
**a** Static contact angle measurements (with error bars) of water, ethylene glycol, and peanut oil on Al alloy surfaces treated with different surface treatments: control (polished), nano-structured (steam-treated), micro-structured (sandblasted), and hierarchical micro-nano structured (SB + ST). The control surface exhibited moderate wettability with the contact angles reducing for the liquids of lower surface tensions. Surface texturing greatly enhanced the performance. The surface hierarchically organized exhibited highest contact angles (> 150° for all liquids), with highest superamphiphobicity. **b **Static contact angle measurements (with error bars) of water, ethylene glycol, and peanut oil on Zn surfaces following various surface treatments: control (polished), nano-structured (steam-treated), micro-structured (sandblasted), and combined micro-nano structured (SB + ST). The control surface had minimal oil repellency, while texturing progressively promoted liquid repellency. The micro-nano structured Zn surface demonstrated contact angles in excess of 150° for all the examined liquids, confirming superamphiphobic performance. Error bars show standard deviations from a minimum of three independent determinations for every sample

As shown in Fig. 3a, the untreated (control) Al alloy surfaces, polished with 5000-grit sandpaper, had moderate hydrophobicity with a 120.7° water contact angle, and rather poor repellency for lower surface tension liquids ~ 108.6° for ethylene glycol and only 89.2° for peanut oil. These results show that the control surface lacked the necessary dual-scale roughness to be able to minimize liquid–solid contact, especially for oils, which spread very easily.

The formation of nano-scale structures by ST significantly enhanced surface repellency. The water contact angle increased to 150.6°, with ethylene glycol and peanut oil angles of 148.2° and 108.8°,

respectively. The formation of dense “nano-grass” on the surface played a key role in forming partial solid–liquid contact, with droplets able to reside on a composite interface of solid and air (Cassie–Baxter state) [33]. However, the failure with peanut oil showed that nano-scale roughness by itself was insufficient to totally repel wetting by low-surface-tension liquids. Sandblasting that produced micro-scale roughness also assisted in inducing wetting resistance. The micro-structured Al alloy surface exhibited water, ethylene glycol, and peanut oil contact angles of 153.2°, 151.9°, and 150.4°, respectively. The flake-like microstructures produced by sandblasting could effectively trap air, and their topography had sufficient curvature to reduce wetting by both polar and non-polar liquids. The most repulsive was recorded on hierarchically organized Al alloy surfaces that were both sandblasted and steam treated (SB + ST), as seen in the micro-nano group. These surfaces had contact angles of 160.04° for water, 156.1° for ethylene glycol, and 154.4° for peanut oil, far beyond the superamphiphobic threshold. The dual-scale surface ensured better air retention and re-entrant geometries, minimizing wetting and promoting droplet mobility. This morphology gives an ideal surface to withstand a wide variety of liquids with diverse surface energies.

#### Wetting performance on zinc surfaces

The Zn substrate also followed the same pattern as illustrated in Fig. 3b. The bare Zn surface offered moderate water resistance 125.8° and ethylene glycol resistance 111.9° but poor resistance to peanut oil 91.5°, indicating a deficiency of roughness to gain omniphobic performance. With nano-scale structuring by ST, the contact angles improved to 158.4° (water), 155.3° (ethylene glycol), and 137.1° (peanut oil). These improvements are explained by the development of short, regular nanowires that disrupted the liquid-solid contact interface. Although nanostructures on Zn were relatively dense in comparison to Al, an increase in nano-scale roughness significantly enhanced repellency, especially for mid-range surface tension liquids. Micro-structured Zn surfaces obtained by SB also exhibited the same repellency increase. Contact angles were 157.2° with water, 154.1° with ethylene glycol, and 148.7° with peanut oil. The random, striated topography offered air trapping within the grooves, with a mechanical barrier effect against liquid intrusion.

Similar to Al alloy, SB + ST-treated Zn surfaces once more exhibited the best performance with 160.1° water, 156.0° ethylene glycol, and 152.8° peanut oil contact angles. Hierarchical micro-nano topography with moderately inclined nanowires anchored in micro-roughened troughs enhanced re-entrant curvature and discouraged capillary intrusion. The comparatively shorter nanowires (compared to those developed in hot water environments) also assisted in the formation of an energetically favorable Cassie–Baxter state.

#### Comparative insights and implications

The consistent increase in contact angles across all test liquids-water, ethylene glycol, and peanut oil on both Al alloy and zinc substrates highlights the critical role of hierarchical micro/nano structuring in achieving true superamphiphobicity. Surfaces with only micro- or nano-scale roughness showed limited repellency, especially against low-surface-tension oils. In contrast, the dual-structured surfaces created by combining sandblasting and ST consistently achieved contact angles above 150°, even for challenging liquids like peanut oil. This demonstrates the effectiveness of the air-retaining surface architecture in resisting wetting and spreading.

These results confirm that the synergy between mechanical abrasion (micro-roughness) and vapor-phase oxidation (nano-roughness) is key to fabricating high-performance superamphiphobic surfaces. The durability and repellency of these coatings make them strong candidates for real-world applications, including self-cleaning materials, anti-smudge finishes, anti-fouling surfaces, and corrosion-resistant coatings in environments exposed to both water- and oil-based contaminants.

### Durability testing of superamphiphobic surfaces

In order to evaluate the utility usability, mechanical and environmental durability of superamphiphobic surfaces developed on Zn and Al alloy substrates were examined. The hierarchical surfaces, which were developed by sandblasting and steam treatment followed by surface energy reduction (SER), were submitted to a series of mechanical and functionality durability tests. They included tape peeling, sandpaper abrasion, water jet impact, self-cleaning, floating, and anti-fogging performance tests. Pre- and post-test contact angle measurement and visual inspection yielded images regarding structural and functional integrity of the developed surfaces.

#### Mechanical resistance: adhesion resistance (Tape peeling Test)

**Fig. 4:**
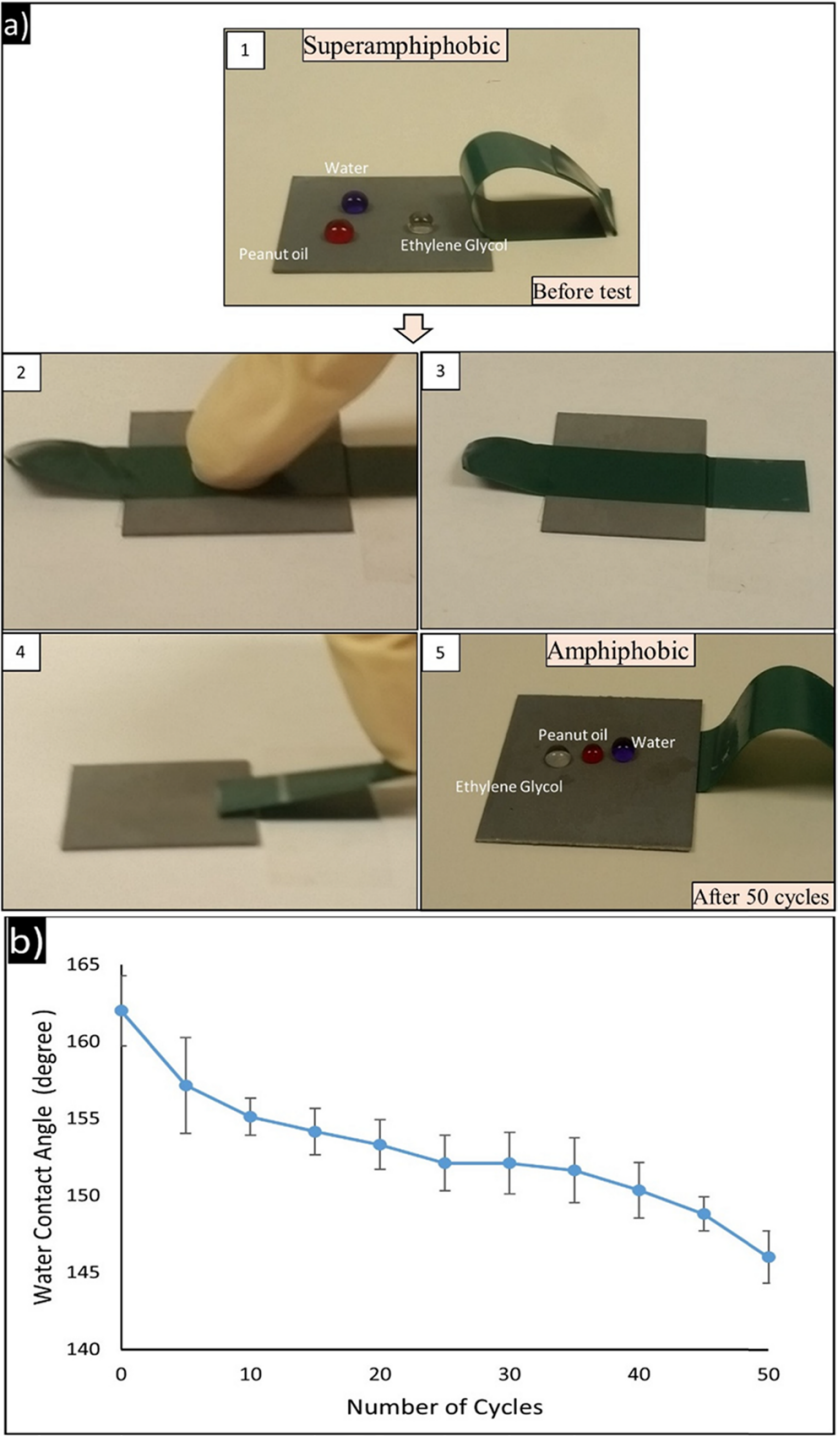
Adhesion resistance (tape peeling test) for superamphiphobic Zn surface. The contact angles before and after 50 cycles of tape adhesion and peeling demonstrate good mechanical durability of the surface. The surface maintains high repellency to ethylene glycol and peanut oil even after repeated peeling, indicating strong interfacial adhesion of the micro/nanostructures

To evaluate the mechanical stability of superamphiphobic surfaces, a tape-peeling test over Zn substrates was conducted, as illustrated in Fig. 4. Prior to this, the obtained surface exhibited 156.1° and 154.4° contact angles for ethylene glycol and peanut oil, respectively, hence confirming its original superamphiphobic status. After 50 cycles of repeated adhesive tape adhesion and peeling, the contact angles reduced to 142.3° and 121.7° for ethylene glycol and peanut oil, respectively.In spite of the mentioned performance loss, surfaces continued to show high liquid repellency, particularly for ethylene glycol. This indicates good interfacial adhesion of the micro-nano structures to the substrate and confirms the durability of the surface functionalization in the face of peeling stress. The decrease in contact angle, particularly for the lower surface tension liquid (peanut oil), is due to localized delamination of the nano-features and rupture of the surface energy-reducing coating. However, the surface was hydrophobic-to-amphiphobic even under mechanical strain and had good resistance to adhesion.

#### Mechanical durability: sandpaper abrasion resistance

**Fig. 5:**
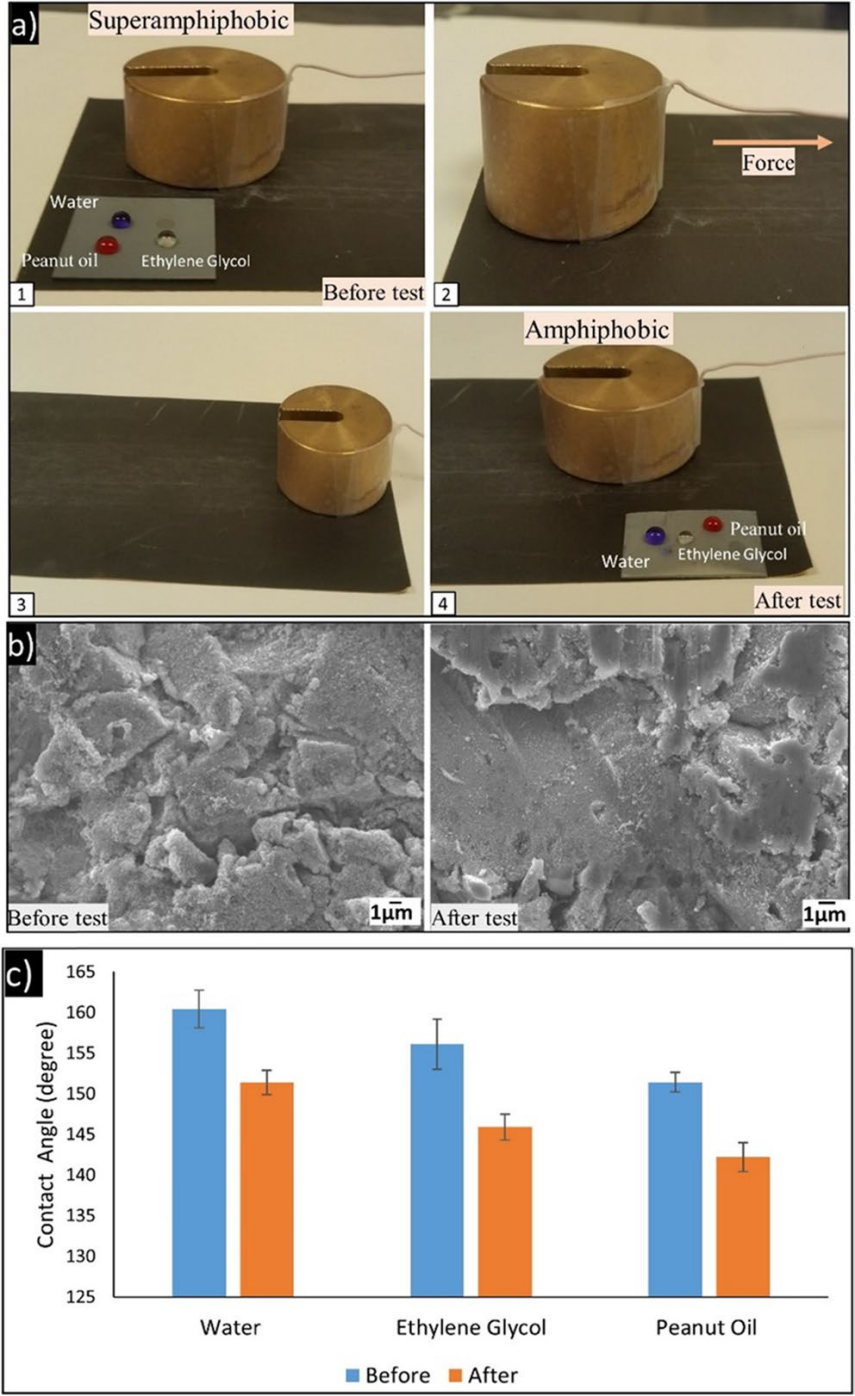
Abrasion resistance of superamphiphobic Zn surface after sandpaper abrasion test. **a** After abrasion under 500 g load over 100 cm on 1500-grit sandpaper, the Zn surface retained significant hydrophobicity and partial hierarchical structuring. This highlights the mechanical robustness imparted by micro/nano dual-scale roughness. **b** SEM image before abrasion, showing intact hierarchical micro/nano structure. SEM image after abrasion, showing partial damage but preservation of roughness sufficient to maintain hydrophobicity. **c** Static water contact angle measurement after water jet exposure, maintaining contact angle >150°. Static ethylene glycol contact angle after abrasion resistance test ~140 o, with minimal change. Static peanut oil contact angle ~ 120 ° showing minor reduction but maintaining amphiphobicity

To simulate mechanical abrasive wear, the superamphiphobic zinc surface was subjected to a sandpaper abrasion test [30, 31]. A 500 g load was applied to the sample, which was then slid over a 1500-grit sandpaper surface for a total distance of 100 cm, replicating conditions of friction and sliding wear, as shown in Fig. 5. Despite a noticeable reduction in performance, the surface retained its hydrophobicity and preserved partial hierarchical structuring. The microscale roughness introduced by sandblasting acted as a mechanical framework, helping to protect the finer nanostructures formed during the nano-textured coatings alone, highlighting the structural advantage of combining micro- and nanoscale features. steam treatment by distributing and deflecting abrasive forces. These findings indicate that the dual-scale surface architecture provides superior mechanical abrasion resistance compared to nano-textured coatings alone, highlighting the structural advantage of combining micro- and nanoscale features.

#### Water jet impact resistance

**Fig. 6:**
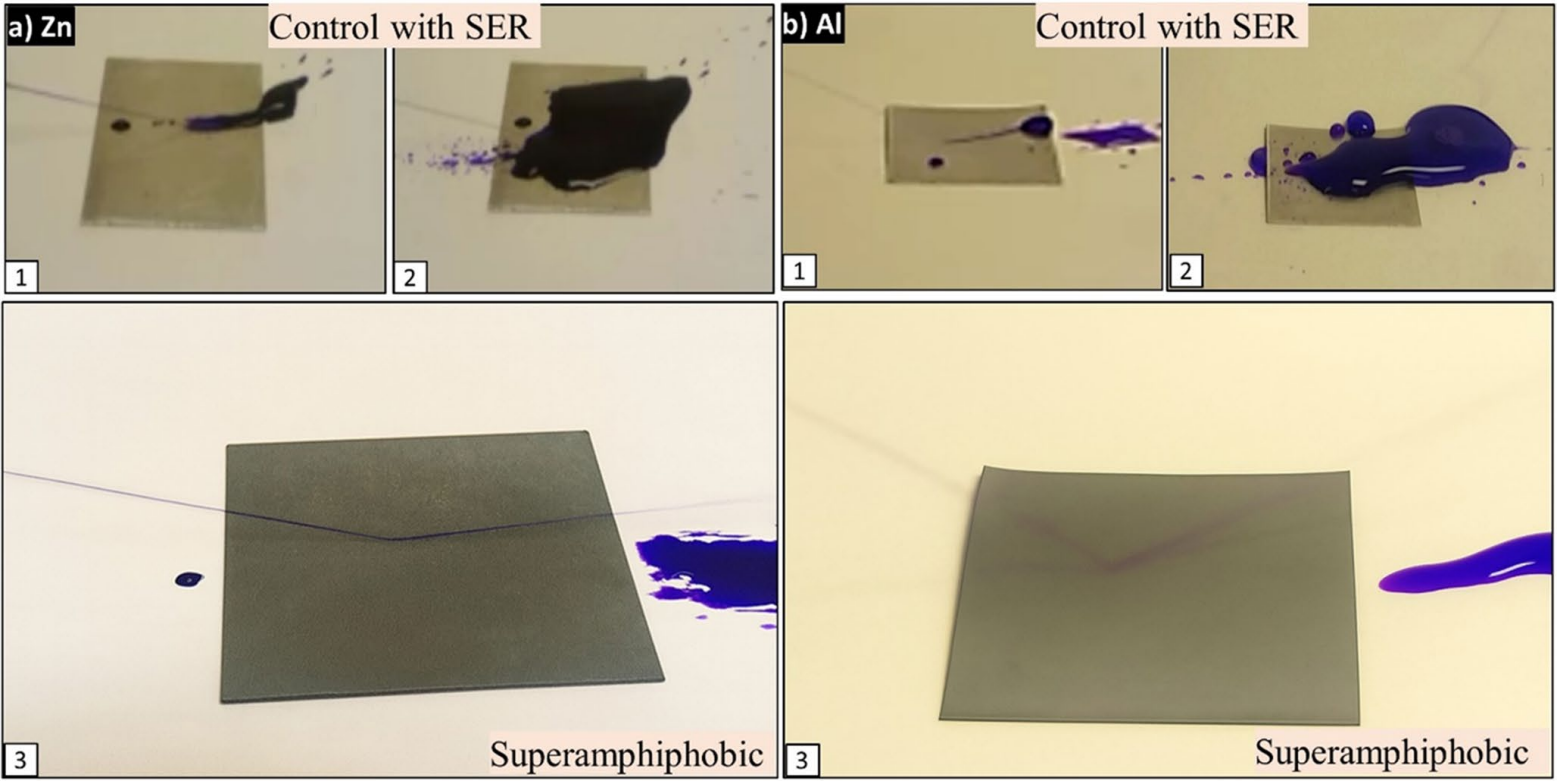
Water jet impact resistance of superamphiphobic **a** Zn and **b** Al alloy surfaces: Following water jet exposure at an angle of ~35° and impact speed of 2.6 m/s, both surfaces maintained high static contact angles (>150° for water and ethylene glycol), confirming resistance to mechanical stress from fluid impact without failure of the Cassie–Baxter state

To probe resistance to mechanical stress due to fluid, a water jet was used to test the superamphiphobic Zn and Al alloy surfaces (Fig. 6a, b). Contact angles after exposure remained greater than 150° for water and ethylene glycol and decreased only slightly for peanut oil. The surfaces were resistant to high-velocity impact from water without failure of the Cassie–Baxter state and maintenance of air pockets beneath the droplet interface. The ability of the hierarchical texture to resist hydrodynamic stress without significant structural failure or wetting transition suggests potential for outdoor, industrial, or marine use where continuous or forceful water exposure is present.

#### Self-Cleaning performance

**Fig. 7:**
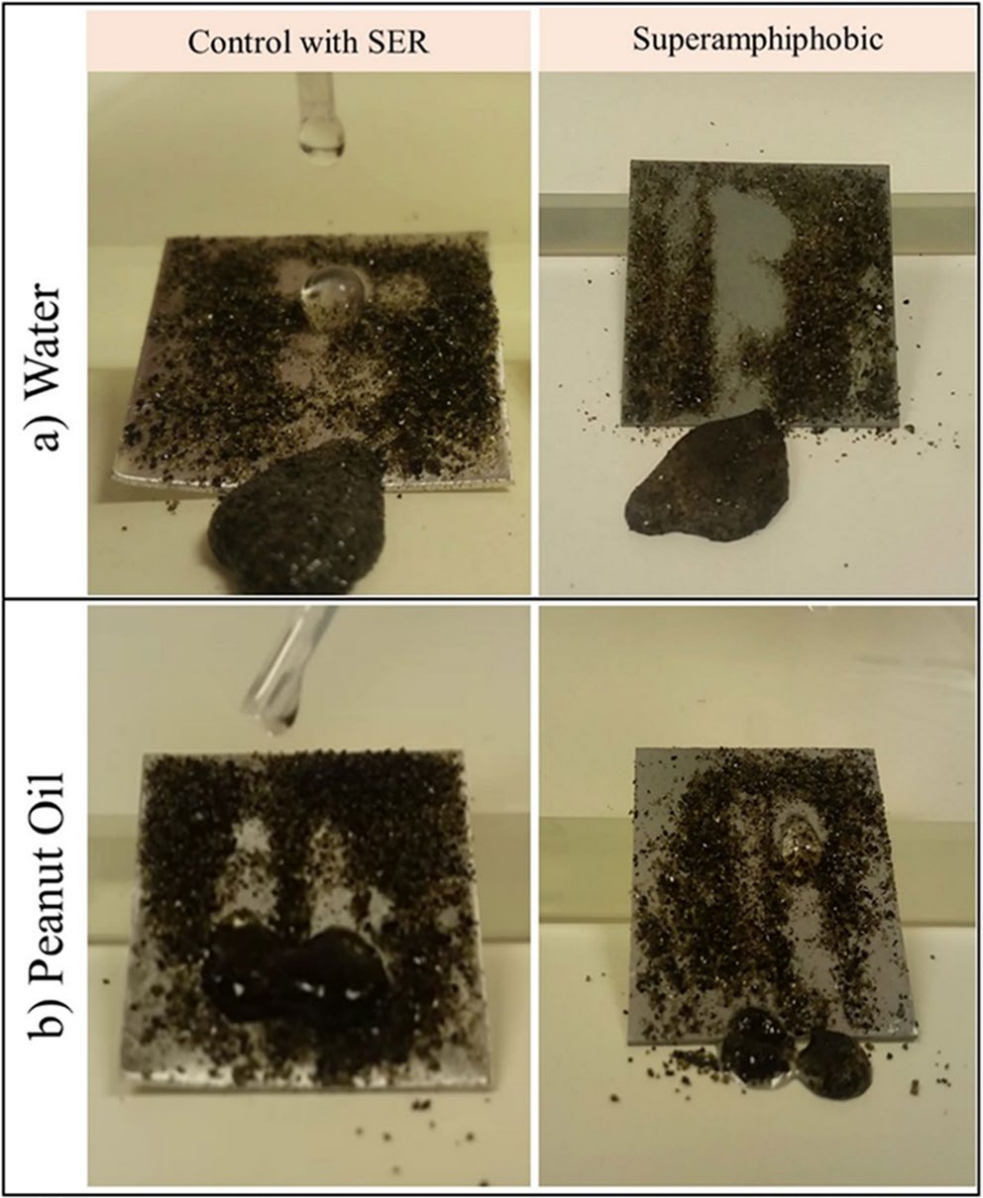
Self-cleaning behavior of dust-contaminated Zn superamphiphobic surfaces: **a** Water droplet rolling off the dusty surface, efficiently removing dust particles. **b** Peanut oil droplet rolling off, confirming low adhesion and excellent self-cleaning

The self-cleaning property of the Zn and Al alloy superamphiphobic surfaces was examined by applying peanut oil and water droplets on dusty samples (Fig. 7a, b). In both cases, droplets effortlessly rolled off, collecting and removing particulate impurities without leaving residue behind. Low-surface-tension oils that rolled off the surface confirm not only the presence of low contact angle hysteresis but also excellent maintenance of surface cleanliness. This property is necessary in reducing fouling and maintenance in real uses such as solar panels, architectural glass, and transport coatings.

#### Liquid flotation test

**Fig. 8:**
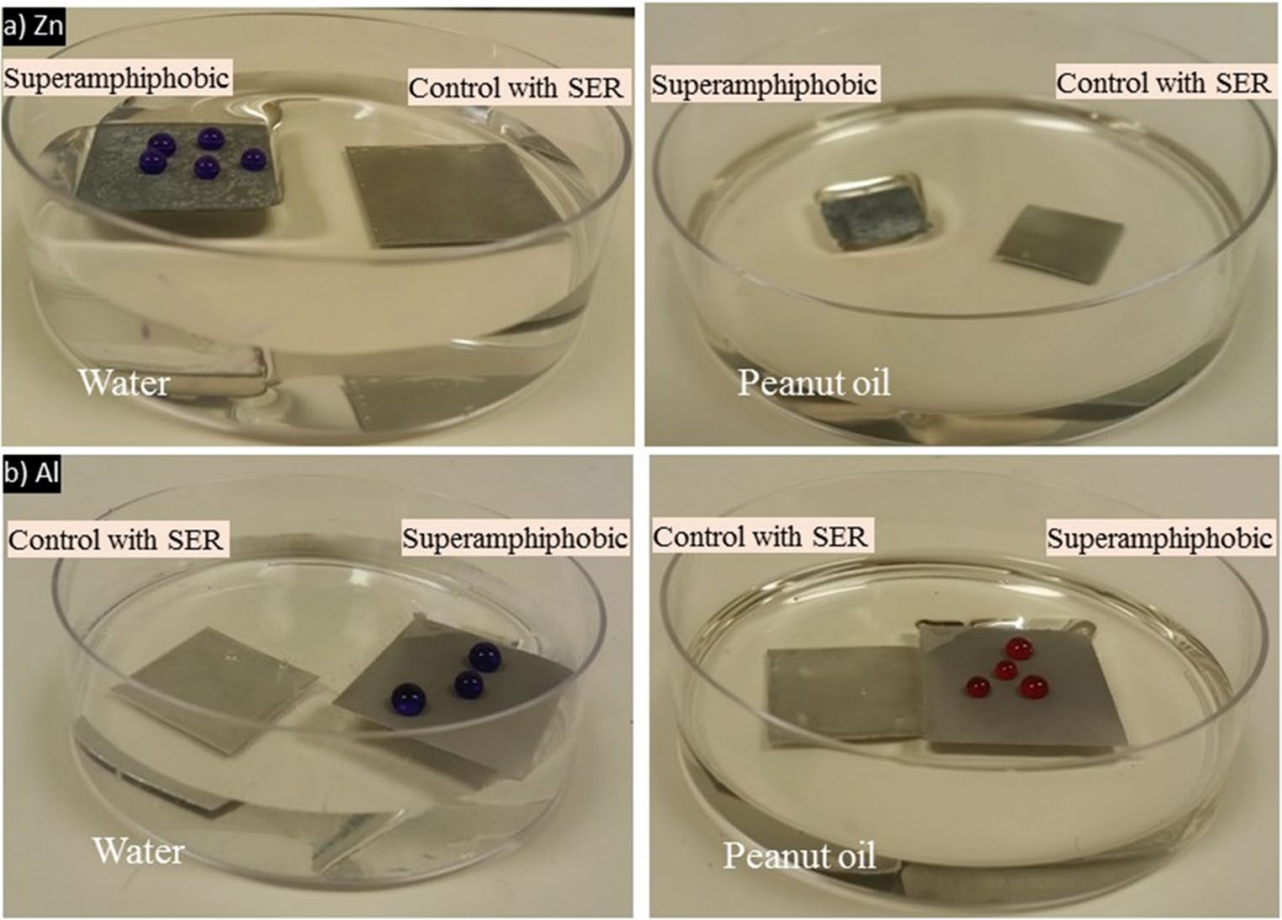
Liquid flotation performance of **a** Zn sample floating on the surface of water without sinking or wetting. Zn sample floating on peanut oil, demonstrating strong omniphobicity, and **b** Al alloy superamphiphobic surfaces: Al sample floating similarly on water and peanut oil surfaces

Liquid flotation tests were performed to visually verify omniphobicity. Placing carefully on the surface of water and peanut oil, Zn and Al alloy samples floated consistently without wetting or sinking (Fig. 8a, b). Floating on oil, a challenging low-surface-tension liquid, demonstrates the surface ability to sustain a metastable Cassie–Baxter regime on polar and non-polar liquids. Such performance reflects ultra liquid repellency and points to the enormous potential of the coating in applications for oil–water separation, anti-biofouling marine surfaces, and floatation-based microfluidic systems.

#### Anti-Fogging functionality

**Fig. 9:**
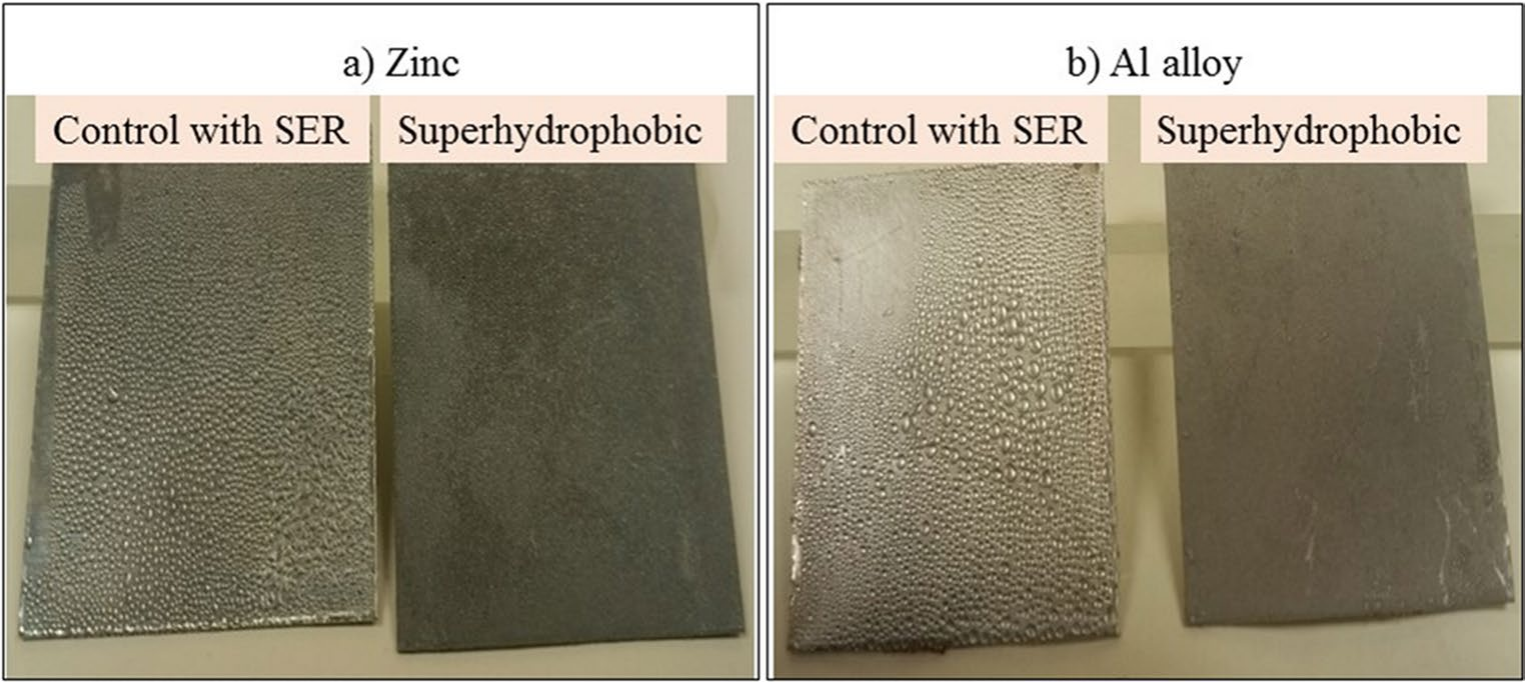
Anti-fogging performance of superamphiphobic **a** Zn and **b** Al alloy surfaces: Control (untreated) sample exposed to humid conditions, showing significant fogging. Treated superamphiphobic sample exposed under the same conditions, remaining clear and fog-free due to suppression of condensation nucleation

To study anti-fogging properties, superamphiphobic Zn and Al alloy samples were both exposed to a humid environment in saturated state (Fig. 9a, b). While control samples became fogged by condensation, treated surfaces remained visually clear to the naked eye. Nano-scale roughness and low surface energy hindered droplet nucleation and coalescence and water film growth [34]. This implies that apart from repelling bulk liquids, the surfaces can also resist water condensation at the microscopic level, and they are thus viable candidates for anti-fog coating on transparent lenses and objects in case of highly humid environments.

## Conclusion

This study presents a low-cost, scalable, and sustainable route for synthesizing mechanically durable superamphiphobic coatings on Al-alloy and Zn substrates using a two-step sandblasting and steam treatment followed by low-surface-energy functionalization. The hierarchical architecture, developed by combining micro-roughness with steam-grown nano-features, induces high repellency toward both water and low-surface-tension liquids (ethylene glycol, and peanut oil) achieving static contact angles greater than 150° and sliding angles below 5°. The films exhibit excellent durability after tape-peeling, abrasive wear, and water-jet testing, while maintaining repeated self-cleaning and anti-fogging performance. The clean, low-chemical process significantly reduces processing time and minimizes the use of toxic reagents compared to conventional chemical etching, offering enhanced durability and broader potential applications. The technique is compatible with common engineering metals (Zn and Al alloys) and holds promise for industrial, outdoor, and marine low-maintenance, anti-fouling, and anti-corrosion coatings. Both steam treatment and sandblasting are scalable steam treatment can be implemented in roll-to-roll or batch systems, and sandblasting in batch or large-area processes. Future engineering optimization may involve adapting sandblasting nozzle parameters, abrasive size, and steam treatment time to ensure homogeneity across complex geometries and large components. Overall, the results confirm that the dual-scale structure provides a robust and viable path toward superamphiphobic coatings that are manufactural and meet key requirements for real-world applications.

## Data Availability

The datasets generated and/or analyzed during the current study are available from the corresponding author upon reasonable request.

## Code Availability

No custom code or software was used in this study. All analyses were performed using standard software and methodologies, as detailed in the manuscript.
